# Antimicrobial peptides from arthropod venoms exhibit activity against *Sporothrix* species

**DOI:** 10.1590/1678-9199-JVATITD-2025-0053

**Published:** 2026-06-19

**Authors:** Maiara Campolina de Miranda, William Gustavo Lima, Izabella Christina de Castro Rodrigues, Felipe Henrique de Souza Silva, Rodrigo Moreira Verly, Julio Cesar Moreira Brito, Maria Elena de Lima, Rachel Basques Caligiorne

**Affiliations:** 1 Graduate Program in Medicine and Biomedicine, Santa Casa de Belo Horizonte School of Medicine, Belo Horizonte, MG, Brazil.; 2 Department of Chemistry, Institute of Exact Sciences, Federal University of the Jequitinhonha and Mucuri Valleys (UFVJM), Diamantina, MG, Brazil.; 3 Graduate Program in Biotechnology, Research and Development Directorate, Ezequiel Dias Foundation (Funed), Belo Horizonte, MG, Brazil.

**Keywords:** Sporothrix brasiliensis, Antimicrobial peptides, Melittin, Membrane disruption, Antifungal synergy, Intralesional formulation, Arthropod venoms

## Abstract

**Background:**

Sporotrichosis is a fungal infection caused by species of the *Sporothrix schenckii* complex. Antifungal treatment with itraconazole and amphotericin B is limited by increasing resistance, adverse effects, and prolonged treatment courses, highlighting the need for novel antifungal strategies.

**Methods:**

The antifungal activity of seven antimicrobial peptides derived from arthropod toxins against *Sporothrix* species was evaluated: six synthetic peptides derived from the spider *Lycosa erythrognatha* toxin and one peptide isolated from apitoxin (melittin). Minimum inhibitory concentrations (MICs) and minimum fungicidal concentrations (MFCs) were determined. Mechanistic assays were performed to investigate membrane damage, oxidative stress induction, and interactions with ergosterol and the fungal cell wall. Synergistic activity with itraconazole was assessed, and the effect of melittin on selected virulence factors was studied. To explore potential therapeutic applications, a melittin-based formulation for local (intralesional) usage was developed, and its cytotoxicity was tested in HEK-293 and HepG2 cell lines, as well as its short-term safety in murine models.

**Results:**

All peptides inhibited *S. schenckii* and *S. brasiliensis*, with MIC and MFC values ranging from 0.5 to 32 µM. Melittin displayed the strongest antifungal effect, acting predominantly through a membranolytic mechanism associated with oxidative stress. Combined with itraconazole, melittin demonstrated synergistic activity against both species. Melittin selectively reduced pyomelanin production, while urease activity remained unaffected. The melittin-based formulation showed lower cytotoxicity compared to melittin alone, and subcutaneous administration in mice was well tolerated at the lowest dose tested (0.1 mg/kg).

**Conclusion:**

Melittin exhibits potent antifungal activity against *Sporothrix* spp. and synergism with itraconazole, supporting further investigation as an antifungal candidate. Although therapeutic efficacy was not evaluated in infected animal models, the development of a safer melittin-based formulation provides a proof-of-concept foundation for future studies focusing on the local treatment of cutaneous sporotrichosis.

## Graphical abstract

**Figure:**
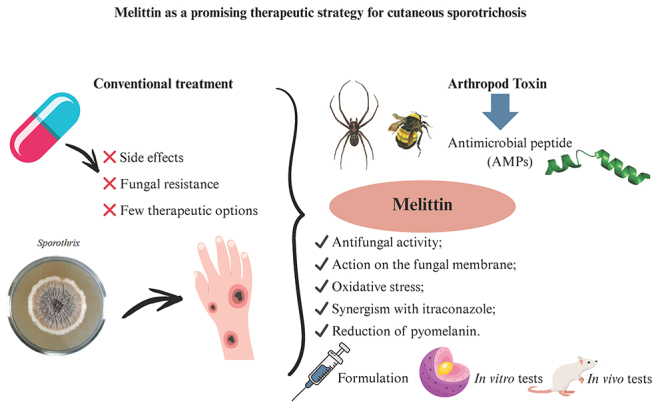

## 1. Background

Sporotrichosis is an infection caused by fungi of the *Sporothrix schenckii* complex (i.e., *S. schenckii, S. brasiliensis, S. globosa, S. luriei, S. albicans,* and *S. mexicana*), which affects humans and other animals, especially domestic cats (*Felis catus*) [1, 2, 3]. These fungi are widely distributed in the environment, notably in soil, plants, and decomposing organic matter, facilitating their dissemination and transmission [2]. The infection is primarily transmitted through traumatic skin inoculation, with risk factors and clinical manifestations varying according to the host’s immune response and individual circumstances, depending on temporal trends and geographic context [1]. Generally, sporotrichosis manifests primarily as cutaneous lesions, which can progress to more severe forms in the absence of appropriate treatment. Although less common, systemic infections can also occur, affecting the lungs, meninges, or joints, particularly in immunocompromised individuals [4].

Fungi of the *Sporothrix* genus exhibit significant zoonotic potential, necessitating appropriate management strategies, novel therapeutic approaches, health education, and public awareness campaigns to mitigate the spread of the disease [1]. Current antifungal treatments for sporotrichosis present considerable limitations, including restricted therapeutic options, emerging antifungal resistance, and narrow therapeutic windows [5]. Although drugs such as itraconazole and amphotericin B are commonly employed, they often exhibit limitations related to efficacy, adverse effects, and prolonged treatment duration. This scenario underscores the need for innovative therapeutic alternatives capable of overcoming the challenges associated with current sporotrichosis pharmacotherapy [2, 5].

In this context, antimicrobial peptides (AMPs) have emerged as a promising strategy for the development of new anti-*Sporothrix* antifungal agents [6, 7]. AMPs offer several advantages over conventional antifungals, including a reduced propensity to induce resistance, minimal residue generation that could exert selective pressure on environmental fungi, a broad antifungal spectrum, potent fungicidal activity, and a rapid onset of action [6, 8]. Although AMPs are found in multiple biological sources, arthropod secretions are particularly noteworthy due to their remarkable structural diversity and biological activity [9]. In this regard, venoms from spiders, scorpions, wasps, bees, and centipedes have been successfully explored as sources of bioactive molecules with activity against medically relevant bacteria, especially multidrug-resistant strains [10]. However, the antifungal potential of arthropod-derived AMPs remains comparatively underexplored, with limited studies focusing on fungal pathogens, including *Sporothrix* spp. [7].

This study evaluated the antifungal efficacy of natural and synthetic peptides derived from two different arthropod venoms - the wolf spider (*Lycosa erythrognatha*) and the honeybee (*Apis mellifera*) - against *Sporothrix* species, aiming to explore their potential as candidates for local sporotrichosis therapy. Furthermore, the *in vitro* and *in vivo* toxicity of a melittin-based formulation was assessed to provide an initial safety profile, supporting its evaluation as a proof-of-concept pharmaceutical approach, which has been registered under patent application number BR1020250249081.

## 2. Methods

### 2.1. Reagents

Itraconazole (Prati-Donaduzzi^TM^, Toledo, PR, Brazil), sodium chloride (NaCl), sorbitol, sodium carbonate (Na_2_CO_3_), sodium bicarbonate (NaHCO_3_), hydrochloric acid (HCl), sodium metabisulfite, L-arginine, L-lysine, glutaraldehyde, ethanol, hematoxylin, eosin (Synth^TM^, São Paulo, SP, Brazil), 3-(4,5-dimethylthiazol-2-yl)-2,5-diphenyltetrazolium bromide (MTT), bovine serum albumin, ergosterol, ascorbic acid, Coomassie brilliant blue G-250 (Sigma Aldrich^TM^, St. Louis, MO, USA), ketamine, xylazine (Syntec^TM^, São Paulo, SP, Brazil), Dulbecco's modified Eagle's medium (DMEM), amphotericin B, penicillin, streptomycin and fetal bovine serum (CultilabTM, Campinas, SP, Brazil) were purchased from commercial suppliers and used without further purification. Sabouraud-dextrose agar supplemented with chloramphenicol (50 mg/L) and brain heart infusion (BHI) broth were purchased from Kasvi^TM^ (São José dos Pinhais, PR, Brazil). Christensen's urea broth was obtained from Urestest^TM^ (Barbacena, MG, Brazil).

All peptides employed in this study are listed in Table 1. The compound LyeTx I, a peptide naturally occurring in the venom of the spider *L. erythrognatha* and also obtained in synthetic form and several of its synthetic analogues (i.e., LyeTx I-b, LyeTx I mn, and LyeTx I mnΔKAc) were purchased from Syn^TM^ (Shanghai, China) and their identity were confirmed using spectrometric technique prior to use. LyeTx I mnΔK and LyeTx I mnΔKwN were obtained through solid-phase synthesis and subsequently purified and characterized at the Chemistry Department of the Universidade Federal dos Vales do Jequitinhonha e Mucuri (Diamantina, MG, Brazil). Melittin was purified from honeybee (*Apis mellifera*) venom according to Lima [8]. All peptides were diluted in autoclaved distilled water, aliquoted into 100 µL microtubes, and stored at -20 °C until use.

**Table 1.** Sequence and physicochemical properties of the peptides used in the study.

Name	ε [M^-1^ cm ^-1^]	Mon.M [Da]	Amino acid residue sequence	Number of residues
LyeTx I	5690	2832,48	**H**-IWLTALKFLGKNLGKHLAKQQLAKL-**NH**_2_	25
LyeTx I-b	5690	2695,34	**Ac**-IWLTALKFLGKNLGKLAKQQLAKL-**NH**_2_	24
LyeTx I mn	5690	1701,11	**H**-IWLTALKFLGKNLGK-**NH**_2_	15
LyeTx I mnΔK	5690	1829,28	**H**-IWLTKALKFLGKNLGK-**NH**_2_	16
LyeTx I mnΔKAc	5690	1871,32	**Ac**-IWLTKALKFLGKNLGK-**NH**_2_	16
LyeTx I mnΔKwN	5690	1829,28	**H**-IWLTKALKFLGKLGK-**NH**_2_	15
Melittin	5690	2847.45	**H**-GIGAVLKVLTTGLPALISWIKRKRQQ	26

ε: Molar extinction coefficient at 280nm; Mon.M: monoisotopic mass.

### 2.2. Microorganisms and cells

Two reference strains obtained from the American Type Culture Collection (ATCC) were included in this study: *S. brasiliensis* MYA 4823^TM^ and *S. schenckii* ATCC 32285^TM^. In addition, ten clinical isolates from human skin lesions, identified through morpho-biochemical and molecular methods by Fernandes [11], were also utilized. For the cytotoxicity assay, a human embryonic kidney epithelial cell line (HEK-293 CRL-1573^TM^) and a hepatocellular carcinoma cell line (HepG2 HB-8065^TM^) were employed.

### 2.3. Antifungal activity

*Minimum inhibitory concentration (MIC)*

The antifungal activity of peptides from *L. erythrognatha* venom (i.e., LyeTx I, LyeTx I-b, LyeTx I mn, LyeTx I mnΔK, LyeTx I mnΔKAc and LyeTx I mnΔKwN) and *A. mellifera* (i.e., melittin) was evaluated by determining the minimum inhibitory concentration (MIC) using the microdilution method according to the Clinical and Laboratory Standards Institute (CLSI) [12], with modifications [13]. A yeast phase inoculum of 10³ colony-forming units (CFU)/mL of *S. schenckii* and *S. brasiliensis*, prepared in brain heart infusion (BHI) broth, was used to fill microplates containing serial dilutions of the peptides (0.25-32 µM). BHI broth was used for MIC determination in all assays due to the slow growth kinetics of *Sporothrix* spp. and to ensure consistent yeast-phase growth under experimental conditions. The plates were then incubated at 37 °C for seven days, and the MIC was determined as the lowest concentration that inhibited visible microbial growth in the wells. The seven-day incubation period was intentionally selected to accommodate the low inoculum (10³ CFU/mL) and the slow growth rate of *Sporothrix* spp., ensuring reliable untreated controls and accurate MIC determination. Itraconazole and amphotericin B were included as positive controls. Reference strains (*S. schenckii* ATCC 32285 and *S. brasiliensis* ATCC MYA-4823) were included as quality controls to validate the susceptibility assays. All experiments were performed in triplicate with at least two independent assays.

*Minimum fungicidal concentration (MFC)*

The fungicidal activity of the compounds was assessed by determining the MFC according to Mathias [13]. Aliquots of 100 µL were taken from wells exhibiting no visible growth in the MIC assay and plated onto the surface of Sabouraud-dextrose agar plates, which were subsequently incubated at 37°C for seven days. The MFC was defined as the lowest concentration that inhibited 99% of colony formation compared to the untreated control. All experiments were performed in triplicate.

### 2.4. Action on fungal cellular membrane

Nucleic acid release (DNA/RNA): The potential of melittin, the most active compound (see Table 2), to induce lysis of *S. schenckii* and *S. brasiliensis* cells was initially assessed by the peptide's ability to promote nucleic acid (DNA/RNA) release. The release of intracellular material, measured by absorbance at 260 nm, was quantified according to Lima [8]. Aliquots of 1 mL from fungal suspensions (10⁶ CFU/mL) prepared in sterile saline solution (0.85% NaCl) were treated with melittin (20 µM). Following incubation for one, two, and seven days, the cells were centrifuged at 1500 *g* at 4 °C for 25 min. The presence of intracellular material in the supernatant was measured using an ultraviolet spectrophotometer (Hitachi U-1100TM, Lancashire, UK) at 260 nm absorbance (an indirect indicator of the presence of DNA/RNA). Amphotericin B (20 µM), an antifungal agent known to lyse *Sporothrix* cells, and untreated cells were used as positive and negative controls, respectively. All experiments were performed in triplicate.

**Table 2.** Minimum inhibitory concentration (MIC) and minimum fungicidal concentration (MFC) of peptides derived from the venoms of *Apis mellifera* (melittin) and *Lycosa erythrognatha* (LyeTx I, LyeTx I-b, LyeTx I mn, LyeTx I mnΔK, LyeTx I mnΔKAc and LyeTx I mnΔKswN) against reference strains of *Sporothrix schenckii* and *Sporothrix brasiliensis.*

Peptides	Origin	Microorganisms			
		*Sporothrix schenkii* ATCC 32285		*Sporothrix brasiliensis* MYA 4823	
		MIC*	MFC*	MIC*	MFC*
Melittin	Venom of *A. mellifera* (N)	0.50 (1.42)	0.50 (1.42)	0.50 (1.42)	0.50 (1.42)
LyeTx I	Venom of *L. erythrognatha* (N)	8 (22.66)	8 (22.66)	8 (22.66)	8 (22.66)
LyeTx I-b	Venom of *L. erythrognatha* (S)	2 (5.39)	2(5.39)	4 (10.78)	4 (10.78)
LyeTx I mn	Venom of *L. erythrognatha* (S)	32 (54.44)	32 (54.44)	32 (54.44)	32 (54.44)
LyeTx I mnΔK	Venom of *L. erythrognatha* (S)	16 (29.27)	16(29.27)	64 (117.07)	64 (117.07)
LyeTx I mnΔKAc	Venom of *L. erythrognatha* (S)	16 (29.94)	16 (29.94)	32 (59.88)	32 (59.88)
LyeTx I mnΔKwN	Venom of *L. erythrognatha* (S)	8 (13.73)	8 (13.73)	8 (13.73)	8 (13.73)
Amphotericin B	Positive control	1.85 (2)	1.85 (2)	1.85 (2)	1.85 (2)
Itraconazole^#^	Positive control	0.02 (0.03)	0.18 (0.25)	0.02 (0.03)	0.18 (0.25)

N: natural peptide whose natural structure has not been modified. S: peptide whose natural structure has been synthetically modified.

*MIC and MFC values are expressed in µM (value outside parentheses) and in µg/mL (value inside parentheses)

^#^ The MIC of itraconazole was determined according to the recommendations of the Brazilian Committee on Antimicrobial Susceptibility (BrCAST), which considered the lowest concentration that leads to 50% inhibition of growth when compared to the antifungal-free growth control.

Protein release: The ability of melittin to induce protein extravasation in *S. schenckii* and *S. brasiliensis* cells was determined by Bradford assay [14]. As previously described, 150 µL of supernatant was mixed with Coomassie brilliant blue G-250 dye and incubated for 2 min. Optical density was measured (OD) at 595 nm (OD595nm) using a spectrophotometer (Bio-Tek Instruments^TM^, Winooski, VT, USA), and the protein concentration (µg/mL) was quantified based on a standard curve generated using bovine serum albumin (2.5-100 µg/mL) (Additional file 1). Amphotericin B (20 µM) and untreated cells served as positive and negative controls, respectively. All experiments were performed in triplicate.

Scanning electron microscopy (SEM): To evaluate morphological alterations in *S. brasiliensis* cells induced by melittin, SEM analysis was performed as described by Lima [15]. Images were acquired using a SEM (Jeol^TM^, Tokyo, Japan), and the morphology of cells treated with melittin (5 µM) was compared with the untreated control. SEM was performed only for *S. brasiliensis* due to its higher clinical relevance and virulence, and because it showed a consistent response to melittin exposure.

### 2.5. Phenotypic effects

Ergosterol binding assay: The binding of melittin in the membrane was investigated using the exogenous ergosterol binding assay as described by Lima [16]. The MIC was determined in the presence of different ergosterol concentrations (50, 100, 200, 300, and 400 µg/mL), and an increase in this value by at least two dilutions was considered indicative of ergosterol binding. Amphotericin B, a drug known to form pores in the fungal membrane by binding to ergosterol, was used as a positive control. Ergosterol was added directly to the BHI broth used for MIC determination.

Sorbitol assay: To assess whether melittin exerts lytic activity by targeting the fungal cell wall, the peptide MIC was determined in the presence of sorbitol (0.8 M), a known osmotic protectant of microbial cell wall. An increase in MIC by at least two dilutions indicated action on the fungal cell wall [16]. Sorbitol (0.8 M) was added directly to the BHI broth used for MIC determination.

Oxidative stress induction assay: Reactive oxygen species (ROS) generation induced by melittin was assessed by determining the MIC after supplementing the BHI broth with ascorbic acid (100 µg/mL), a known antioxidant [17]. An increase in MIC by at least two dilutions after the addition of ascorbic acid was considered presumptive evidence of oxidative damage. Itraconazole, an agent known to induce oxidative stress in fungal cells, served as a positive control. 

### 2.6. Effect on virulence

Urease inhibition: A suspension of a hypervirulent clinical isolate of *S. brasiliensis* (*S. brasiliensis* 484) [11], was exposed to subinhibitory concentrations of melittin (corresponding to MIC, ½ MIC, and ¼ MIC: 0.250; 0.125, and 0.06 µM, respectively) for seven days and subsequently incubated in Christensen's urea broth. Urease activity generates a red-colored product, and it was quantified by measuring absorbance at 559 nm using a spectrophotometer (Bio-Tek Instruments^TM^, Winooski, VT, USA) after seven days of incubation at 37 °C. The results obtained were compared with those of the untreated control [18].

Melanin inhibition: The hypervirulent clinical isolate used in the previous assay was cultured in BHI broth supplemented with melittin at ½ MIC (0.25 µM) for seven days. Following this period, the cells in suspension were evaluated spectrophotometrically at 310 nm (Hitachi U-1100TM, Lancashire, UK), which indicates the presence of insoluble melanin isoforms (1,8-dihydroxynaphthalene (DHN)-melanin and eumelanin). Pyomelanin, a soluble isoform, was quantified in the supernatant following culture centrifugation (3000 g at 4 °C for 25 min) by measuring the optical density at 340 nm using an ultraviolet spectrophotometer (Hitachi U-1100TM, Lancashire, UK) [19].

### 2.7. Melittin and itraconazole synergism

The synergistic activity of melittin in combination with itraconazole, the first-line treatment for human and animal sporotrichosis, was evaluated using the checkerboard assay, as described by Lima [16]. Synergy was assessed by calculating the fractional inhibitory concentration (FIC) index according to Equation 1:

FIC_index_ (FICI) = FIC_Itraconazole_ + FIC_Melittin_

Where,

FIC_Itraconazole_ = MIC_Itraconazole combined_/MIC_Itraconazole only_

FIC_Melittin_ = MIC_Melittin combined_/MIC_Melittin only_

According to Oroojalian [20], the FICI values indicate whether the effect is synergistic (FICI≤0.5), additive (0.5>FICI≥1), indifferent (1>FICI≥4) or antagonistic (FICI>4).

### 2.8. Formulation for intralesional use

Due to the promising antifungal activity of melittin, we aimed to develop a formulation for intralesional application in cases of cutaneous sporotrichosis. Five diluents (distilled water, 0.9% NaCl, 0.2% HCl, 5% Na_2_CO_3_, and 5% NaHCO_3_) and three antioxidant agents (L-lysine 75 mg/mL, L-arginine 75 mg/mL, and sodium metabisulfite 0.6%) were evaluated. The two excipients from each class (i.e., diluent and antioxidant) that demonstrated the most favorable performance in MIC assays against *S. brasiliensis* ATCC MYA 4823^TM^, after the addition of the active pharmaceutical ingredient (melittin), were selected for inclusion in the final formulation.

### 2.9. Formulation toxicity

**2.9.1. In vitro *toxicity***

The cytotoxicity of the formulation and the active pharmaceutical ingredient (melittin) were evaluated *in vitro* using MTT assay [21]. Mammalian cells were cultured in 75 cm^3^ flasks containing DMEM supplemented with fetal bovine serum (5%), L-glutamine (50 mg/mL), and an antimicrobial solution (0.3% penicillin-streptomycin-amphotericin B solution at 10,000 U/mL + 10 mg/mL + 2 mg/mL, respectively). For the cytotoxicity assay, the cells were seeded into microplates at a density of 25,000-30,000 cells per well, followed by the addition of 100 μL of DMEM containing serial dilutions of either the formulation or melittin (0.5-128 μM). The plates were incubated at 37 °C in an incubator with a 5% CO_2_ atmosphere for 24 h. Cell viability was then determined using the MTT colorimetric assay [21]. The cytotoxic concentration for 50% cell death (CC_50_) was calculated using cell viability [22].

To assess how many times the compound is more toxic to pathogens than to mammalian cells, the selectivity index (SI) was calculated. This was achieved by dividing the MIC values of the formulation or melittin, obtained in the antimicrobial evaluation assays against *S. brasiliensis* ATCC MYA4823^TM^, by the CC_50_ values of each compound/formulation in kidney and liver cells [23].

**2.9.2. In vivo *toxicity***

All experimental procedures were conducted in accordance with internationally recognized principles for ethical laboratory animal handling. The study protocol was previously approved by the Animal Research Ethics Committee of Santa Casa BH Hospital (CEUA 01/2023). The *in vivo* formulation toxicity was investigated according to the Organization for Economic Co-operation and Development (OECD) acute toxicity protocol [24], with modifications. Fifteen female BALB/c mice (4-5 weeks old; weighing approximately 20 g) were used. Animals were administered subcutaneously with injections of a blank formulation or a formulation-containing melittin at three different doses (0.1, 1, and 10 mg/kg). A saline (NaCl 0,9%) treated group served as a control. Each experimental group consisted of three animals. Ten microliters of each solution were injected into the right hind paw pad with 72-hour intervals for 14 consecutive days. Following the 14-day period, mice were anesthetized (ketamine 60 mg/kg and xylazine 8 mg/kg; intraperitoneal), and blood was collected by brachial plexus exsanguination. Animals were then euthanized by cervical dislocation. Collected blood was stored in tubes with ethylenediaminetetraacetic acid (EDTA) on ice. After euthanasia, necropsies were performed to collect the liver, kidneys, heart, lungs, and brain. The doses (0.1, 1, and 10 mg/kg) were included to explore a preliminary safety window and were determined based on MIC values and previous toxicological data for melittin, considering local (intralesional) application.

Biometric and dietary factors: Before group allocation, the initial body mass of the mice (g) was recorded on the first day using a digital scale (SF-400^TM^, São Paulo, SP, Brazil). The amount of food offered (g) and water volume (mL) provided were measured using a calibrated test tube. After 14 days, the animals were weighed, and food and water consumption were measured. Macronutrient intake was calculated based on the nutritional composition provided on the food label (Labina^TM^, Goiânia, GO, Brazil).

Paw edema: Paw thickness was measured using a caliper (MTK-5000^TM^, São Paulo, SP, Brazil) before injections. Paw thickness was reassessed 24 hours after the initial injection. Edema was quantified as the change in paw thickness (mm), subtracting the baseline measurement from the post-treatment measurement. Additionally, photographs of the paws were taken to visually assess volumetric differences between groups.

Macroscopic, microscopic, and functional changes in target organs: The liver, kidneys, spleen, heart, lungs, and brain were excised and weighed before and after drying in an incubator at 37 °C for 24 hours. The difference between the masses was used as an indirect indicator of organ edema [25]. Subsequently, plasma was obtained by centrifuging the collected blood at 3,000 *g* for 20 minutes. Plasma alanine aminotransferase (ALT), aspartate aminotransferase (AST), and creatinine levels were evaluated using commercial kits according to the manufacturer's instructions (Bioclin^TM^, Belo Horizonte, MG, Brazil). For histological analysis, liver and kidney samples were fixed in 10% buffered formalin, dehydrated in xylene, processed, and embedded in paraffin. Sections (4 µm thick) were cut, fixed on glass slides, and stained with hematoxylin and eosin. Histological analysis was performed using a light microscope (Carl Zeiss AGTM, Oberkochen, B-W, Germany) at 200x or 400x magnifications by a qualified pathologist.

### 2.10. Statistical analysis

Data normality was assessed using the Shapiro-Wilk test in SPSS Statisticsv.19 software. For normally distributed data, a one-way analysis of variance (One-way ANOVA) was performed, followed by two post-tests: Dunnett'sto compare with the control group and Tukey's comparison test analysis to compare differences between concentrations. All statistical analyses were evaluated using GraphPad Prism 5.03 (GraphPad Software Inc.TM, LaJolla, CA) and p-values less than 0.05 were considered statistically significant.

## 3. Results

The antifungal activity of peptides derived from *L. erythrognatha* and *A. mellifera* venoms was initially evaluated by determining the MIC and MFC against *S. schenckii* ATCC 32285 and *S. brasiliensis* MYA 4823. As shown in Table 2, melittin exhibited the highest activity against both *S. schenckii* and *S. brasiliensis*, with a MIC of 0.50 µM (1.42 µg/mL). This cationic peptide demonstrated fungicidal activity against both species, with an MFC of 0.50 µM (1.42 µg/mL). Based on this superior antifungal performance (MIC/MFC), melittin was selected for subsequent mechanistic, virulence, synergy, formulation, and safety investigations.

Among the peptides derived from *L. erythrognatha* venom, the prototype LyeTx I demonstrated significant activity against both *S. schenckii* and *S. brasiliensis*, with a MIC of 8 µM (22.66 µg/mL). The removal of the histidine residue at position 16 (LyeTx I-b) resulted in a two-fold reduction in the MIC against *S. schenckii* (2 µM or 5.39 µg/mL) compared to LyeTx I. A similar trend was observed with *S. brasiliensis*, although the MIC reduction was only one dilution (4 µM or 10.78 µg/mL). The N-terminal fragment of LyeTx I (LyeTx I mn), comprising the first 15 amino acid residues, retained activity against both *S. schenckii* and *S. brasiliensis*, with a MIC of 32 µM (54.44 µg/mL), suggesting the importance of this region for the antifungal effect of LyeTx I. The addition of a lysine residue to LyeTx I mn (i.e., LyeTx I mnΔK) exhibited species-specific effects. While LyeTx I mnΔK showed a lower MIC against *S. schenckii* (16 µM or 29.27 µg/mL) compared to LyeTx I mn, its activity against *S. brasiliensis* was reduced (64 µM and 117.07 µg/mL). Acetylation of the N-terminal region of the LyeTx I mnΔK did not alter its antifungal activity against *S. schenckii* (16 µM or 29.94 µg/mL) but resulted in a one-dilution reduction in the MIC against *S. brasiliensis* (32 µM or 59.88 µg/mL). Furthermore, the removal of the asparagine residue from LyeTx I mnΔK (LyeTx I mnΔKwN) enhanced the antifungal effect against both species, resulting in a one- and two-fold reduction in the MIC against *S. schenckii* and *S. brasiliensis* (8 µM or 13.73 µg/mL for both species), respectively, compared to LyeTx I mnΔK. The peptides derived from the *L. erythrognatha* toxin were fungicidal, as revealed by the equivalent MIC and MFC values observed in all cases (Table 2).

Melittin activity was subsequently evaluated against ten human clinical isolates of *S. brasiliensis* (Table 3). Melittin exhibited MIC values ranging from 0.12 µM (0.35 µg/mL) to 2.00 µM (5.69 µg/mL) against these clinical isolates, demonstrating activity in all cases.

**Table 3.** Minimum inhibitory concentration (MIC) and minimum fungicidal concentration (MFC) of melittin and positive controls (amphotericinB and itraconazole) against different human clinical isolates of *S. brasiliensis*.

Microorganism	Melittin		Amphotericin B		Itraconazole^#^	
	MIC*	MFC*	MIC*	MFC*	MIC*	MFC*
*S. brasiliensis* IC219	2.00 (5.69)	2.00 (5.69)	0.93 (1.00)	0.93 (1.00)	≤ 0.18 (≤ 0.25)	≤ 0.18 (≤ 0.25)
*S. brasiliensis* IC253	1.00 (2.85)	1.00 (2.85)	0.93 (1.00)	0.93 (1.00)	≤ 0.18 (≤ 0.25)	0.72 (1.00)
*S. brasiliensis* IC417	0.50 (1.42)	0.50 (1.42)	≤ 0.23 (≤ 0.25)	≤ 0.23 (≤ 0.25)	≤ 0.18 (≤ 0.25)	≤ 0.18 (≤ 0.25)
*S. brasiliensis* IC441	0.50 (1.42)	0.50 (1.42)	1.85 (2.00)	1.85 (2.00)	0.36 (0.50)	0.72 (1.00)
*S. brasiliensis* IC467	1.00 (2.85)	1.00 (2.85)	0.93 (1.00)	0.93 (1.00)	≤ 0.18 (≤ 0.25)	≤ 0.18 (≤ 0.25)
*S. brasiliensis* IC484	0.50 (1.42)	0.50 (1.42)	3.72 (4.00)	3.72 (4.00)	≤ 0.18 (≤ 0.25)	≤ 0.18 (≤ 0.25)
*S. brasiliensis* IC517	1.00 (2.85)	1.00 (2.85)	0.47 (0.50)	0.47 (0.50)	≤ 0.18 (≤ 0.25)	≤ 0.18 (≤ 0.25)
*S. brasiliensis* IC522	1.00 (2.85)	1.00 (2.85)	0.93 (1.00)	0.93 (1.00)	≤ 0.18 (≤ 0.25)	0.36 (0.50)
*S. brasiliensis* IC540	0.12 (0.35)	0.12 (0.35)	≤ 0.23 (≤ 0.25)	≤0.23 (≤0.25)	≤ 0.18 (≤ 0.25)	≤ 0.18 (≤ 0.25)
*S. brasiliensis* IC541	1.00 (2.85)	1.00 (2.85)	0.93 (1.00)	0.93 (1.00)	≤ 0.18 (≤ 0.25)	0.36 (0.50)

*MIC and MFC values are expressed in µM (value outside parentheses) and in µg/mL (value inside parentheses).

^#^ The MIC of itraconazole was determined according to the recommendations of the Brazilian Committee on Antimicrobial Susceptibility (BrCAST), which considered the lowest concentration that leads to 50% growth inhibition when compared to an antifungal-free growth control.

**Figure 1.**
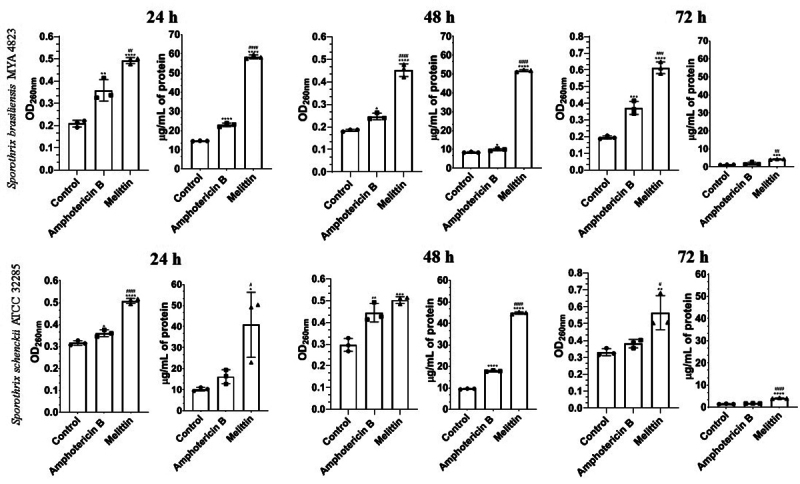
Membranolytic effect of melittin (20 µM) on *Sporothrix* spp. evaluated by the release of nucleic acids (DNA/RNA; absorbance at 260 nm) and proteins. Untreated cells were used as negative controls, and amphotericin B (20 µM) as a positive control. Data are presented as the mean ± SD of representative time points. Statistical analysis was performed using one-way ANOVA followed by Tukey’s post-test. *p* < 0.05; *p < 0.01; **p < 0.001; ***p < 0.0001 versus control; #*p* < 0.05; ##*p* < 0.01; ###*p* < 0.001; ####*p* < 0.0001 versus amphotericin B.

Antimicrobial peptides are frequently associated with membranolytic mechanisms, thus, the effect of melittin on the *Sporothrix* membrane was investigated using DNA/RNA and protein release assays. As shown in Figure 1, melittin caused cell lysis in both *S. schenckii* and *S. brasiliensis*, surpassing amphotericin B at all evaluated time points. Figure 1 displays representative time points (24 h, 48 h, and seven days); additional assessed time points are not shown. In *S. schenckii*, melittin triggered a sustained release of 260 nm-absorbing materials for up to seven days, whereas amphotericin B exhibited this effect for only 48 hours, suggesting a more prolonged effect of the peptide compared to the positive control. Furthermore, in the protein release assay with *S. schenckii*, amphotericin B exhibited a slower effect than melittin. Specifically, the polyene induced protein extravasation only after 48h of incubation, while melittin promoted this effect after 24 hours of exposure (Figure 1).

**Figure 2.**
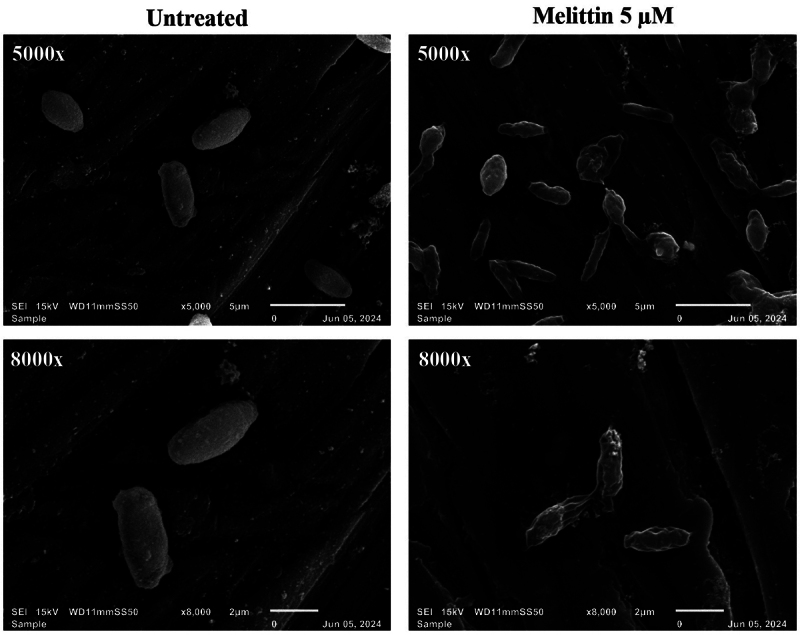
Ultrastructural effects of melittin on *Sporothrix brasiliensis* MYA 4823 cells observed by scanning electron microscopy. Untreated cells exhibit fusiform morphology and an intact cell surface. Cells exposed to melittin (5 µM) show surface irregularities, membrane projections, pore formation, reduced cell volume, and the leakage of intracellular content.

To examine the effect of melittin on the structure of fungal cells, SEM was performed on *S. brasiliensis* MYA 4823 treated with 5 µM melittin. As shown in Figure 2, melittin induced significant morphological changes on the surface of fungal cells, including plasma membrane projections, pores, asymmetric divisions, and the release of cytoplasmic content. Compared to untreated cells, melittin exposure also resulted in cell volume reduction, numerous surface irregularities, and the collapse of cellular structures. These findings confirm that melittin has a potent membranolytic activity against *Sporothrix*.

**Figure 3.**
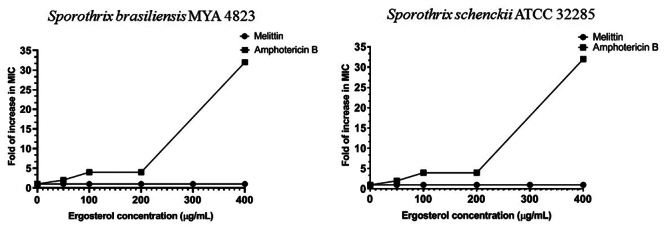
Effect of exogenous ergosterol (50-400 µg/mL) on the minimum inhibitory concentration (MIC) of melittin and amphotericin B against *Sporothrix schenckii* ATCC 32285 and *Sporothrix brasiliensis* MYA 4823.

To elucidate the mechanism by which melittin induces cell lysis in *Sporothrix*, several experiments were conducted. To investigate whether melittin binds to ergosterol in fungal membrane, a mechanism observed with polyene antifungals, the melittin MIC was determined in the presence of increasing ergosterol concentrations (50-400 µg/mL). As shown in Figure 3, the melittin MIC remained unchanged in the presence of exogenous ergosterol, suggesting that this peptide does not interact with ergosterol in the pathogen membrane. In contrast, amphotericin B exhibited an increase in MIC in the presence of exogenous ergosterol in a concentration-dependent manner in both, *S. brasiliensis* and *S. schenckii*, validating the experimental conditions. Next, the potential involvement of the fungal cell wall in melittin's lytic mechanism, a target of echinocandin antifungals, was investigated by determining the MIC in the presence of sorbitol (0.8 M), a known osmotic fungal cell wall protector. As presented in Table 4, no alteration in the melittin MIC was observed, indicating that the peptide does not target the cell wall of either *S. brasiliensis* or *S. schenckii*. Finally, the contribution of oxidative stress to melittin activity was assessed by adding ascorbic acid (100 µg/mL), an antioxidant, to the growth medium. As indicated in Table 4, the melittin MIC increased by one and three dilutions for *S. brasiliensis* and *S. schenckii*, respectively. This result shows that oxidative stress plays a role, at least in part, in the mechanism of the melittin lytic effect. Itraconazole, a known pro-oxidant drug, exhibited a significant reduction in activity upon the addition of ascorbic acid to the medium, further validating the experimental conditions.

**Table 4.** Minimum inhibitory concentration (MIC) of melittin against *Sporothrix* species in the absence or presence of sorbitol (0.8 M) or ascorbic acid (100 µg/mL).

Compounds	***S. brasiliensis* MYA 4823**			***S. schenckii*ATCC 32285**		
	Control	+/Sorbitol	+/Ascorbic acid	Control	+/Sorbitol	+/Ascorbic acid
Melittin	0.50	0.50	1.00	0.50	0.50	4.00
Itraconazole	0.03	-	2.00	0.03	-	> 32

**Figure 4.**
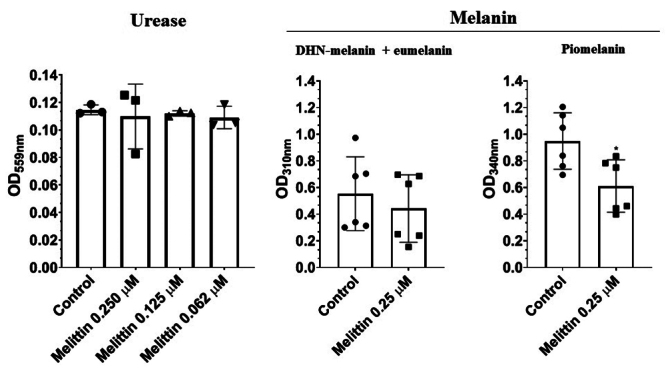
Effect of subinhibitory concentrations of melittin (MIC, ½ MIC, and ¼ MIC) on urease activity and melanin production by a hypervirulent clinical isolate of *Sporothrix brasiliensis*. Data were analyzed using one-way ANOVA followed by Dunnett’s post-test. *p* < 0.05 versus control.

The effect of melittin on *S. brasiliensis* virulence factors was evaluated by its activity on urease and melanin production in a hypervirulent strain (isolate 484). Initially, the effect of melittin on urease activity was assessed using Christensen's urea broth. According to Figure 4, melittin did not inhibit urease production or activity. Subsequently, the production or release of *S. brasiliensis* melanin varieties was examined after exposure to a subinhibitory melittin concentration. Treatment with this peptide modulated melanin levels in *Sporothrix*, as evidenced by the reduced production or excretion of the soluble isoform pyomelanin (Figure 4).

Given the frequent use of combined antifungal therapies, which are usually more effective than monotherapy, the interaction between melittin and itraconazole, the first-line sporotrichosis treatment, was investigated using the checkerboard assay. The results demonstrated a synergistic interaction between melittin and itraconazole against *S. schenckii* (FICI 0.27) and *S. brasiliensis* (FICI 0.50) (Table 5).

**Table 5.** Fractional inhibitory concentration (FIC) and FIC index (FICI) of melittin in combination with itraconazole against *Sporothrix schenckii* and *Sporothrix brasiliensis.*

Microorganisms	FIC		FICI	Effect
	Melittin	Itraconazole		
*Sporothrix schenckii* ATCC 32285	0.25	0.02	0.27	Synergistic
*Sporothrix brasiliensis* MYA4823	0.25	0.25	0.50	Synergistic

FICI: fractional inhibitory concentration index. The FICI was interpreted as follows: FICI ≤ 0.5 indicates synergistic effect; 0.5 > FICI ≥ 1.0 indicates additive effect; 1.0 > FICI ≥ 4.0 indicates indifferent effect; FICI > 4.0 indicates antagonistic effect.

For formulation development, various diluents (sodium carbonate, sodium bicarbonate, neutral saline, saline acidified with hydrochloric acid) and antioxidant agents (L-arginine, L-lysine, sodium metabisulfite) were evaluated to determine the optimal combination. According to Table 6, 5% sodium bicarbonate (NaHCO_3_) proved to be the most effective diluent, even reducing the MIC of melittin by three dilutions against *S. brasiliensis* MYA 4823 (MIC 0.06 µM). L-arginine emerged as the most suitable antioxidant, reducing the melittin MIC by one dilution (MIC 0.25 µM). Consequently, the final formulation was prepared by combining 5% sodium bicarbonate with L-arginine (75 mg/mL), followed by the incorporation of melittin. Although the final formulation exhibited a three-dilution reduction in antifungal activity compared to the solution with melittin alone, it still retained significant biological activity against *S. brasiliensis*, with a MIC of 4 µM.

**Table 6.** Minimum inhibitory concentration (MIC; µM) of formulations containing or not melittin against *Sporothrix brasiliensis* MYA 4823.

Excipients	MIC formulation without API	MIC formulation with API
**Solvents**		
Distilled water	> 128	0.50
NaCl 0.9%	> 128	2.00
NaCl 0.9% + HCl 0.2%	> 128	> 128
Na_2_CO_3_ 5%	> 128	4.00
NaHCO_3_ 5%	> 128	0.06
**Antioxidants**		
L-lysine 75 mg/mL	> 128	4.00
L-arginine 75 mg/mL	> 128	0.25
Sodium metabisulfite 0.6%	> 128	2.00
**Formulation**		
NaHCO_3_ 5% + L-arginine 75 mg/mL	> 128	4.00

API: active pharmaceutical ingredient (melittin).

**Figure 5.**
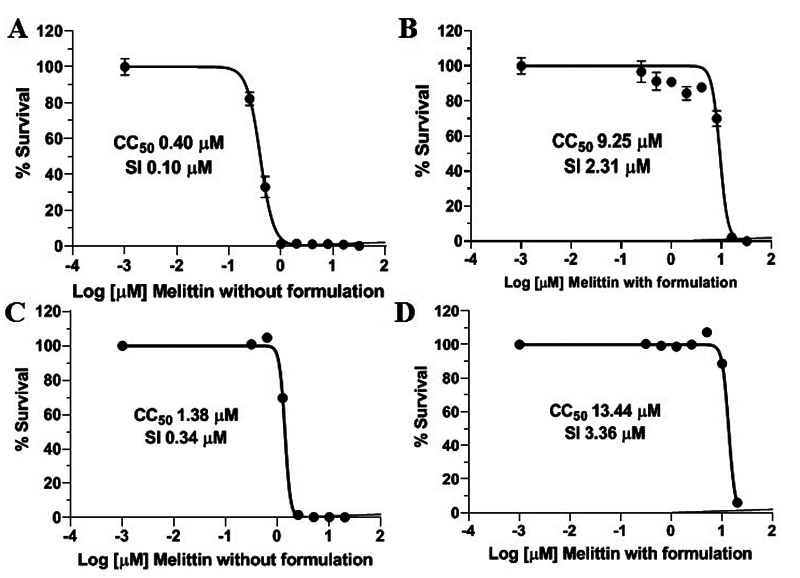
Cytotoxic concentration for 50% of cells (CC50) and selectivity index (SI) of melittin alone and the melittin-based formulation against: **(A, B)** HEK-293 human kidney cell lines; and **(C, D)** HepG2 human liver cell lines.

After evaluating the *in vitro* efficacy of the developed formulation, its *in vitro* and *in vivo* safety was assessed. Initially, formulation cytotoxicity was determined using an MTT assay with kidney (HEK-293) and liver (HepG-2) cell lines. As demonstrated in Figure 5, melittin exhibited a substantial reduction in cytotoxicity after formulation. The cytotoxic concentration for 50% of the cells (CC_50_) increased from 0.40 µM to 9.25 µM in kidney cells and from 1.38 µM to 13.44 µM in liver cells. To evaluate the reduction in toxicity achieved through formulation, the selectivity index (SI) of melittin alone and in the formulation, was calculated as the ratio between the CC_50_ values for mammalian cells and the MIC against *S. brasiliensis* MYA 4823. The results revealed that melittin alone was approximately 20% more toxic to kidney cells than to *S. brasiliensis*. However, after formulation, it was 2.3 times more toxic to the pathogen compared to kidney cells. Similarly, in liver cells, melittin alone was 10% more toxic than to *Sporothrix* isolates, but this effect was reversed in the formulation, with melittin becoming 3.36 times more selective for the pathogen relative to HepG2 cells.

Following the *in vitro* safety assessment, the formulation toxicity was evaluated after subcutaneous administration in BALB/c mice. Animals receiving either the blank formulation or the formulation with melittin (0.1, 1, and 10 mg/kg) showed no difference in weight gain compared to the control group (Table 7). However, an increase in food and macronutrient (lipid, protein, and carbohydrate) intake, was observed in the groups receiving the formulation containing melittin, especially in animals treated with the lowest dose (0.1 mg/kg). Water intake, conversely, was reduced in all animals that received the formulation, regardless of the presence of melittin (Table 7).

**Table 7.** Dietary behavior of animals in the acute toxicity test of the formulation containing melittin.

Variable	Groups				
	Control	Blank formulation	Melittin 0.1 mg/kg	Melittin 1 mg/kg	Melittin 10 mg/kg
Δ Weight (g)	1.50±2.08	1.50±1.00	2.75±0.50	1.75±1.50	2.00±0.82
Food intake (g/g of animal)	6.77±0.57	7.35±0.46	11.17±0.48****	7.79±0.44**####	7.91±0.34**####
Water intake (mL/g of animal)	22.91±1.93	13.29±0.84***	12.81±0.55***	12.75±0.89***	11.65±0.50***
Proteins (mg/g of animal)	1557±131.10	1690±106.90	2570±110.70****	1793±99.74**####	1818±78.30**####
Lipids (mg/g of animal)	270.80±22.81	293.90±18.59	446.90±19.25****	311.80±17.35**####	316.20±13.62**####
Carbohydrates (mg/g of animal)	3047±256.60	3306±209.10	5028±216.50****	3507±195.10**####	3558±153.20**####
Caloric intake (kcal/g of animal)	20.85±1.76	22.63±1.43	34.41±1.48	24.00±1.34	24.35±1.05
Feed efficiency index (%)	23.50±31.05	20.39±13.39	24.65±4.63	23.93±22.15	25.33±10.40

An asterisk (*) represents a statistical difference with 0.05 ≤ p-value > 0.01 compared to the control group. Four asterisks (****) represent a statistical difference with p-value ≤ 0.0001 compared to the control group. Four hash marks (^####^) represent a statistical difference with p-value ≤ 0.0001 compared to the melittin 0.1 mg/kg group. Data normality was assessed by the Shapiro-Wilk test. The results were then compared by One-Way ANOVA with Tukey's post-test.

**Figure 6.**
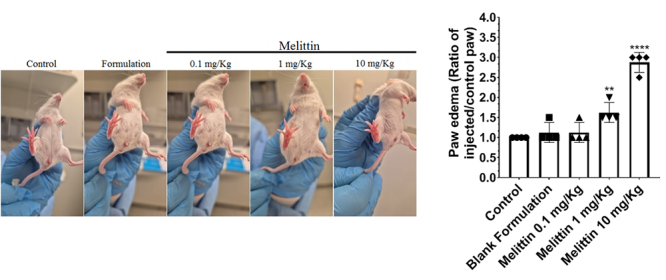
Paw edema induced by the subcutaneous (intraplantar) administration of blank formulation or the melittin-containing formulation (0.1, 1, and 10 mg/kg). Animals treated with a 0.9% saline solution were used as controls. Data were analyzed using one-way ANOVA followed by Dunnett’s post-test. *p < 0.01; ***p < 0.0001 *versus* control.

**Figure 7.**
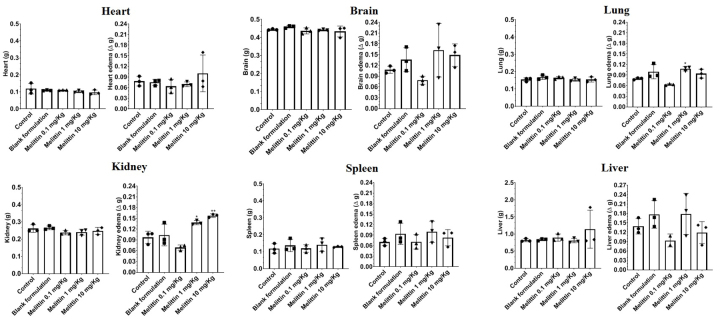
Weight variation and edema of target organs (heart, brain, lung, kidney, spleen, and liver) in animals treated with the blank formulation or the melittin-containing formulation (0.1, 1, and 10 mg/kg). Saline-treated animals were used as controls. Data were analyzed using one-way ANOVA followed by Dunnett’s post-test. *p* < 0.05; *p < 0.01 *versus* control.

Paw size revealed that the formulation containing melittin induced significant paw edema at the highest concentrations tested (1 and 10 mg/kg) (Figure 6). Furthermore, edema analysis in target organs revealed that the 1 mg/kg melittin formulation induced edema in the lungs and kidneys, while the 10 mg/kg dose induced only pulmonary edema (Figure 7). Importantly, no toxic effects were observed with the 0.1 mg/kg formulation, suggesting that at this concentration, the formulation is safe.

**Figure 8.**
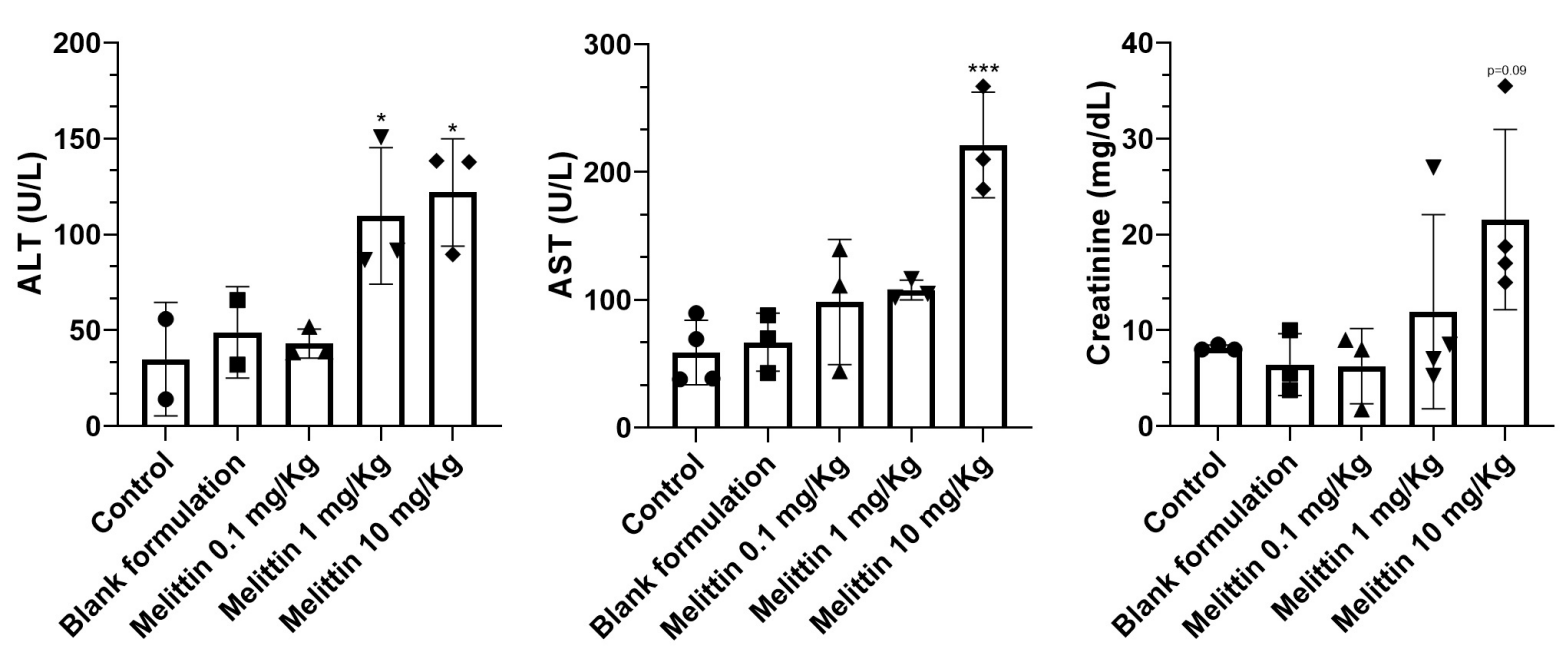
Serum levels of alanine aminotransferase (ALT), aspartate aminotransferase (AST), and creatinine in animals treated with the blank formulation or the melittin-containing formulation (0.1, 1, and 10 mg/kg). Saline-treated animals were used as controls. Data were analyzed using one-way ANOVA followed by Dunnett’s post-test. *p* < 0.05; *p < 0.01 versus control.

**Figure 9.**
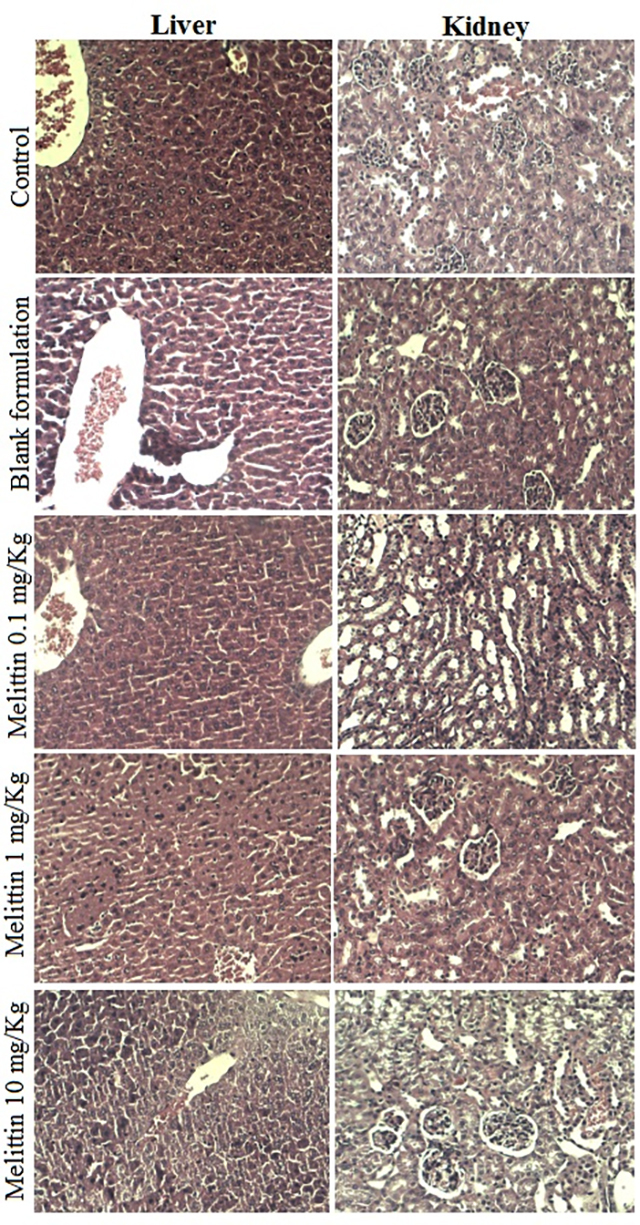
Representative histological sections of the liver and kidney from animals treated with the developed formulation. Images were obtained at 400× magnification.

Finally, the effects of the formulation on liver and kidney microstructure and function were studied. As shown in Figure 8, animals that received the formulation containing melittin at a dose of 1 mg/kg exhibited an increase in the liver enzyme alanine aminotransferase (ALT), while those receiving the 10 mg/kg formulation showed elevations in both ALT and aspartate aminotransferase (AST), suggesting liver toxicity at these doses. These dose-dependent findings were interpreted in relation to the *in vitro* melittin antifungal potency (MIC range 0.12-2.00 µM for clinical isolates; MIC 0.50 µM for reference strains) and to the intended local (intralesional) application, highlighting that higher systemic exposure levels (1-10 mg/kg) are associated with increased toxicity compared to the lowest tested dose (0.1 mg/kg). Kidney function, assessed by measuring creatinine levels, revealed a slight increase in animals treated with 10 mg/kg melittin (p-value = 0.09). Subsequently, liver and kidney structures were examined through histological analyses. Figure 9 demonstrates that only animals that received 10 mg/kg melittin exhibited structural changes in these organs. An increase in cellularity was observed in the liver of animals treated with the 10 mg/kg formulation, suggesting hepatitis. Glomerular atrophy, with an increase in the Bowman's spaces of the glomerulus, was observed in the kidneys.

## 4. Discussion

Given the rise in antifungal resistance and treatment challenges in sporotrichosis, identifying safer and more effective therapies is essential [26]. In this proof-of-concept study, we evaluated the antifungal activity of arthropod toxin-derived peptides against different *Sporothrix* species, investigating their efficacy and toxicity, and exploring a pharmaceutical formulation intended for local (intralesional) application in cutaneous sporotrichosis.

Antimicrobial peptides have been widely investigated as potential antifungal agents, including against species of the *Sporothrix* genus [27, 28]. Among the peptides with demonstrated activity against the etiological agent of sporotrichosis, ToAP2d (FIKRIARLLRKIF; 1681.08 Da) [29, 30] and gallerimycin (VDKPPYLPRPRPPRRIYNR-NH₂; 2431.42 Da) are notable [31]. ToAP2d exhibits significant antifungal activity against *S. globosa*, promoting fungal cell apoptosis and stimulating the immune response in murine models [29, 30]. Gallerimycin, identified in the wax moth larva (*Galleria mellonella*), also has an antifungal effect against *S. brasiliensis*, suggesting its action in the arthropod's innate immune response [31]. These peptides demonstrate promising antifungal activity, highlighting the relevance of AMPs in the development of new therapeutic approaches against sporotrichosis.

**Figure 10.**
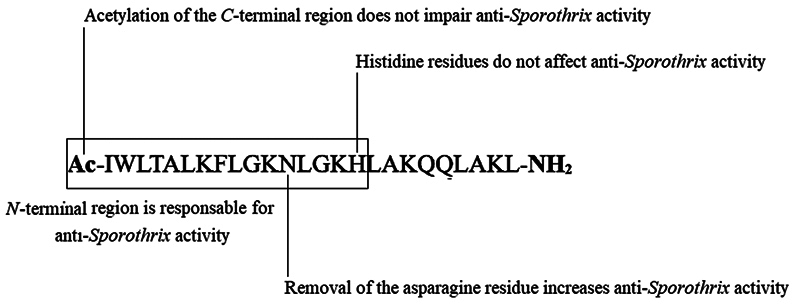
Structure-activity relationship (SAR) analysis of peptides derived from the LyeTx I toxin.

Among the peptides evaluated in this study, derived from the toxins of the spider *Lycosa erythrognatha* (LyeTx I, LyeTx I-b, LyeTx I mn, LyeTx I mnΔK, LyeTx I mnΔKAc and LyeTx I mnΔKwN) and the honeybee *Apis mellifera* (melittin), melittin demonstrated the best *in vitro* results against *S. schenckii* and *S. brasiliensis*. An analysis of the structure-activity relationship of the peptides derived from the *L. erythrognatha* toxin highlights that the N-terminal region is the pharmacophore of this compound family. Additionally, we revealed that the histidine at position 16 does not impact the anti-*Sporothrix* activity, and acetylation does not impair the antifungal activity (Figure 10). This profile is relevant because the addition of acetyl groups reduces degradation by endogenous peptidases, increasing AMP stability *in vivo* [32]. 

Furthermore, we showed that the removal of the asparagine residue increases the anti-*Sporothrix* effect of these peptides (Figure 10). On the other hand, melittin exhibits activity against other medically important fungi, such as *Candida albicans*, *Aspergillus* spp., *Trichophyton beigelii,* and *Botrytis cinerea* [33, 34]. However, the antifungal effect of melittin against *Sporothrix* pathogens had not been previously elucidated. Therefore, this is the first study to identify the biological activity of melittin against the sporotrichosis pathogen. Furthermore, the MFC results confirmed the fungicidal action of melittin against *Sporothrix*. Fungicidal substances are strong candidates for clinical use due to their ability to reduce the likelihood of disease complications in severe cases and to accelerate clinical and microbiological cure [8]. Nevertheless, we emphasize that the present findings support an early-stage evaluation of melittin as an antifungal candidate rather than immediate clinical translation.

The fungicidal effect of melittin on *Sporothrix* can be attributed to membranolytic mechanism. Compounds that lyse fungal cells, such as echinocandins and amphotericin B, are known for their potent fungicidal activity [35]. Exposing *Sporothrix* to melittin resulted in intracellular content leakage (DNA/RNA and proteins) and numerous ultrastructural membrane alterations, confirming its membranolytic effect on this microorganism. Picoli et al. [36] have confirmed that melittin induces lysis, destabilizing cell membranes and promoting the release of cytoplasmic content. Its primary antimicrobial mechanism involves plasma membrane permeabilization through pore formation, which allows the passage of ions, resulting in membrane potential dissipation and the extravasation of intracellular components [37]. Furthermore, melittin induces the production of intracellular reactive oxygen species (ROS), leading to oxidative damage and lipid peroxidation, ultimately culminating in cell death via apoptosis [38]. These findings corroborate our results, which demonstrated that ascorbic acid reduced the antifungal activity of this peptide against *Sporothrix*, confirming its pro-oxidant effect. The membranolytic and pro-oxidant actions of melittin are advantageous as they reduce the likelihood of resistance development, since resistance mechanisms involving membrane alterations impose a high biological cost on the pathogen [39]. We also highlight that the oxidative contribution observed in the ascorbic acid assay indicates a partial mechanistic component and should be interpreted as supportive, rather than definitive, evidence of oxidative damage.

The treatment of animal and human sporotrichosis often involves combinations of different antifungals due to the enhanced therapeutic effects observed with the simultaneous use of multiple drugs [3]. Therefore, we investigated the interaction between melittin and itraconazole, the first-line drug for sporotrichosis therapy, and observed synergistic interactions between these two compounds against both *S. schenckii* and *S. brasiliensis*. This synergism can be attributed to their complementary action mechanisms [40]. While itraconazole inhibits ergosterol synthesis, compromising the fungal plasma membrane structure, melittin directly destabilizes the cell membrane through pore formation and increased permeability [41, 42]. This enhanced permeability not only compromises fungal cellular integrity but also facilitates the entry of other substances, such as itraconazole [22]. These data suggest that combination strategies may allow dose reduction and improve antifungal performance; however, confirmation of therapeutic benefit requires *in vivo* efficacy studies.

Multiple virulence factors have been identified in *Sporothrix*, particularly in *S. brasiliensis*, which is recognized as the most pathogenic species. Urease, for example, is directly involved in the ability of *Sporothrix* to resist adverse conditions, promoting tissue invasion and playing a critical role in modulating the host's immune response [43, 44]. Melanins, in turn, are potent antioxidant agents that protect the fungus from oxidative stress induced by physical, chemical, and biological factors, and play an important role in resistance to antifungal agents [19, 45]. Our results demonstrated that melittin exposure did not inhibit urease production or activity in a hypervirulent *S. brasiliensis* strain, but reduced the production or excretion of pyomelanin, a soluble melanin isoform. Thus, the impact of melittin on virulence-related traits appears selective and partial rather than broad-spectrum, and interfering with pyomelanin production may potentially decrease the fungal ability to resist oxidative defenses, supporting the observed pro-oxidant effect in the ascorbic acid assay.

In this study, it was observed that melittin exhibits significant cytotoxicity in HEK-293 kidney cells (CC50 0.40 µM) and in HepG2 liver cells (CC50 1.38 µM). These findings corroborate previous studies reporting high melittin toxicity in liver, lung, bladder, kidney, prostate, and breast mammalian cell lines [46]. However, the developed formulation, combining 5% sodium bicarbonate (NaHCO₃) and L-arginine as excipients, effectively reduced melittin toxicity. The selectivity indices confirm that the formulation increased the selectivity of melittin for fungal cells while decreasing its toxicity in human cells.

Once the *in vitro* toxicity reduction was observed, the formulation's effect was evaluated after subcutaneous use in healthy animals. The results showed that formulations containing melittin at 1 mg/kg and 10 mg/kg exhibited systemic toxicity, with evidence of hepatotoxicity and nephrotoxicity. However, the 0.1 mg/kg melittin formulation demonstrated its safety for *in vivo* use, with no signs of local or systemic toxicity. Although melittin is a potent antimicrobial agent, its clinical use faces challenges due to its low cellular selectivity [47]. According to Saeed and Khalil [48], the maximum sublethal dose of melittin in BALB/c mice is 2.4 mg/kg, and the median lethal dose (LD_50_) is 4.96 mg/kg. However, Gui et al. [49] demonstrated that this peptide does not cause significant cumulative toxicity; showing that repeated intraperitoneal administration of non-toxic doses did not result in significant renal or hepatic damage and reduced blood glucose levels in diabetic animals. These findings suggest the potential use of low and repeated doses. Therefore, the developed 0.1 mg/kg of melittin formulation appears promising in this context.

It is important to highlight that the apparent disconnect between *in vitro* potency (MIC values in the submicromolar-to-low micromolar range) and *in vivo* tolerability at higher systemic doses should be interpreted within the intended clinical context. The present *in vivo* study was designed to provide an initial safety assessment of repeated administration in healthy animals and does not establish pharmacokinetic equivalence between MIC values and tissue concentrations. Since this study aimed to suggest a local (intralesional) treatment of cutaneous sporotrichosis, achieving higher concentrations at the lesion site with limited systemic exposure may be feasible; however, defining a true therapeutic index will require pharmacokinetic analyses and therapeutic efficacy testing in infected models.

This study has important limitations that must be acknowledged. Firstly, the formulation was not therapeutically evaluated in an infected animal model; therefore, conclusions regarding *in vivo* efficacy against sporotrichosis cannot be drawn at this stage. Secondly, long-term and cumulative toxicity were not assessed beyond the experimental window, and additional studies are needed to define safety after prolonged exposure. Thirdly, although our mechanistic assays support membranolytic activity and suggest an oxidative contribution, they should be considered supportive rather than definitive. Taken together, the current results provide a robust proof-of-concept foundation, while further studies in infected models, including pharmacokinetic and long-term safety evaluations, are essential to advance clinical translatability.

## 5. Conclusion

The results of this study indicate that some AMPs, such as those obtained from the spider *Lycosa erythrognatha* and melittin from *Apis mellifera*, showed promising activity against sporotrichosis. However, melittin seems to be the most active agent and represents a potential prototype for the development of novel antifungal therapies against cutaneous sporotrichosis. This peptide demonstrated a low fungicidal concentration, a membranolytic effect, and the capacity for synergistic interaction with itraconazole. Furthermore, its ability to inhibit the production/excretion of pyomelanin, a virulence factor contributing to *Sporothrix* resistance, offers significant potential therapeutic benefits in the treatment of sporotrichosis. Despite its potent antifungal effects, the toxicity of melittin limits its clinical application. However, the intralesional formulation developed in this study effectively reduces its toxicity, offering a promising option for human and veterinary cutaneous sporotrichosis.

### Abbreviations

%: percentage; ×g: relative centrifugal force; °C: degrees Celsius; µg/mL: microgram per milliliter; µL: microliter; µM: micromolar; ALT: alanine aminotransferase; AMPs: antimicrobial peptides; AST: aspartate aminotransferase; ATCC: American Type Culture Collection; BHI: brain heart infusion; CC50: 50% cytotoxic concentration; CEUA: Ethics Committee on Animal Use; CFU/mL: colony-forming units per milliliter; CLSI: Clinical and Laboratory Standards Institute; DMEM: Dulbecco’s modified Eagle’s medium; DNA: deoxyribonucleic acid; EDTA: ethylenediaminetetraacetic acid; FIC: fractional inhibitory concentration; FICI: fractional inhibitory concentration index; h: hours; HCl: hydrochloric acid; HEK-293: human embryonic kidney cell line; HepG2: human hepatocellular carcinoma cell line; LD50: median lethal dose; mg/kg: milligram per kilogram; mg/L: milligram per liter; mg/mL: milligram per milliliter; min: minutes; mL: milliliter; mm: millimeter; MTT: 3-(4,5-dimethylthiazol-2-yl)-2,5-diphenyltetrazolium bromide; Na_2_CO_3_: sodium carbonate; NaCl: sodium chloride; NaHCO₃: sodium bicarbonate; nm: nanometer; OD: optical density; OECD: Organization for Economic Co-operation and Development; RNA: ribonucleic acid; ROS: reactive oxygen species; SEM: scanning electron microscopy; SI: selectivity index; spp.: species; U/mL: units per milliliter.

## Supplementary material

The following online material is available for this article:

Additional file 1. Analytical curve and equation of the line employed to determine protein concentration in the cell lysis assay.

## Availability of data and materials 

 All data generated or analyzed during this study are included in this published article (and its additional files).
