# Supplementary material for: Antimicrobial peptides from arthropod venoms exhibit activity against Sporothrix species
Source: J Venom Anim Toxins Incl Trop Dis. 2026 Jun 19;32:e20250053. doi: 10.1590/1678-9199-JVATITD-2025-0053 (PMC13285926; doi:10.1590/1678-9199-JVATITD-2025-0053)
Supplement: Additional file 1. [file 1678-9199-jvatitd-32-e20250053-s1.pdf]

J Venom Anim Toxins incl Trop Dis

https://doi.org/10.1590/1678-9199-JVATITD-2025-0053

Supplementary Material to "Antimicrobial peptides from arthropod venoms exhibit activity against *Sporothrix* species"

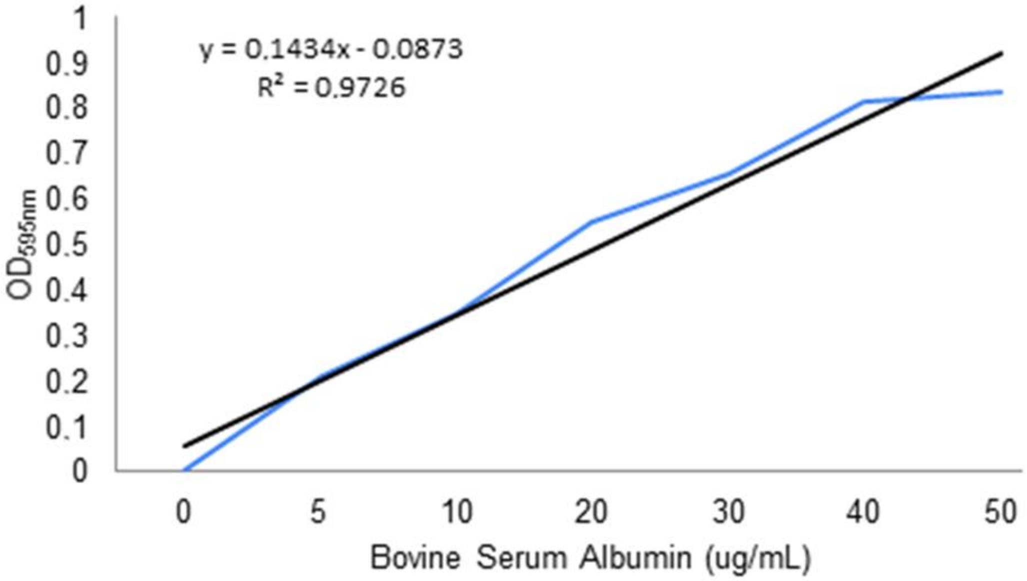

Additional file 1. Analytical curve and equation of the line employed to determine protein concentration in the cell lysis assay.
